# Kidney whole-transcriptome profiling in primary antiphospholipid syndrome reveals complement, interferons and NETs-related gene expression

**DOI:** 10.1093/rheumatology/keae397

**Published:** 2024-08-06

**Authors:** Maria G Tektonidou, Kleio-Maria Verrou, Harikleia Gakiopoulou, Menelaos Manoloukos, Panagiotis Lembessis, Pantelis Hatzis, Petros P Sfikakis

**Affiliations:** First Department of Propaedeutic and Internal Medicine, Joint Academic Rheumatology Program, ‘Laiko’ General Hospital, School of Medicine, National and Kapodistrian University of Athens, Athens, Greece; First Department of Propaedeutic and Internal Medicine, Joint Academic Rheumatology Program, ‘Laiko’ General Hospital, School of Medicine, National and Kapodistrian University of Athens, Athens, Greece; Centre of New Biotechnologies and Precision Medicine (CNBPM), School of Medicine, National and Kapodistrian University of Athens, Athens, Greece; First Department of Pathology, School of Medicine, National and Kapodistrian University of Athens, Athens, Greece; Centre of New Biotechnologies and Precision Medicine (CNBPM), School of Medicine, National and Kapodistrian University of Athens, Athens, Greece; Centre of New Biotechnologies and Precision Medicine (CNBPM), School of Medicine, National and Kapodistrian University of Athens, Athens, Greece; Centre of New Biotechnologies and Precision Medicine (CNBPM), School of Medicine, National and Kapodistrian University of Athens, Athens, Greece; Institute for Fundamental Biomedical Research, Biomedical Sciences Research Center Alexander Fleming, Vari, Greece; First Department of Propaedeutic and Internal Medicine, Joint Academic Rheumatology Program, ‘Laiko’ General Hospital, School of Medicine, National and Kapodistrian University of Athens, Athens, Greece; Centre of New Biotechnologies and Precision Medicine (CNBPM), School of Medicine, National and Kapodistrian University of Athens, Athens, Greece

**Keywords:** antiphospholipid syndrome, nephropathy, innate immunity, interferon, complement, neutrophils

## Abstract

**Objective:**

Pathogenesis of antiphospholipid syndrome (APS) remains poorly elucidated. We aimed to evaluate for the first time kidney transcriptome profiles in primary APS *vs* systemic lupus erythematosus (SLE) and control subjects.

**Methods:**

We performed RNA sequencing on archival formalin-fixed paraffin-embedded kidney biopsies from APS (*n* = 4), SLE (*n* = 5) and control (*n* = 3) individuals, differential gene expression analysis (DGEA) and enrichment analysis using gene ontology (GO) and CORUM, KEGG and Reactome pathway databases.

**Results:**

Two-dimensional projection showed a distinct gene profile in primary APS *vs* control kidneys samples, but similar to SLE. DGEA in APS *vs* controls returned 276 upregulated and 217 downregulated genes, while the comparison between APS and SLE identified 75 upregulated and 111 downregulated genes. In 276 upregulated genes, enriched GO terms were (innate) immune response, inflammatory response, leucocyte and lymphocyte activation, cytokine production and T cell activation. CORUM and KEGG revealed complement-related genes (C3, C4A, C4B). Expression levels showed logFC values of 2.25 (*P* = 1.58e-05) for C3, 2.17 (*P* = 2.69e-06) for C4A and 2.135 (*P* = 3.7e-06) for C4B in APS *vs* controls, without differences between APS and SLE. Interferon (IFN) alpha/beta signalling was revealed by Reactome. Expression levels of nine IFN-regulated genes found upregulated in APS *vs* control kidneys (*P*-values ≤ 0.001 for all). Examining neutrophil-extracellular traps (NETs)-related gene expression, 13 of 15 upregulated NETs-related genes exhibited higher expression in APS *vs* controls but not *vs* SLE.

**Conclusion:**

Complement, interferon and NETs-related genes are highly expressed in APS kidney tissues, similarly to SLE, pointing out the role of innate immunity in APS nephropathy pathogenesis and potential treatment targets.

Rheumatology key messages

- This is the first RNA sequencing study on kidney biopsies of primary APS patients.
- Complement, interferon and NETs-related genes are highly expressed in APS kidney tissues, supporting the role of innate immunity in APS.
- Aberrant innate immunity responses in APS pathogenesis underscores potential treatment targets.

## Introduction

Antiphospholipid syndrome (APS) is a systemic autoimmune disorder with life-threatening complications and poorly understood pathophysiology. Increasing evidence from experimental studies has shed light on the role of thromboinflammation in macrovascular/thrombotic APS [1, 2]. However, the pathogenesis of APS nephropathy and other APS microvascular manifestations, recently included in the 2023 ACR/EULAR classification criteria for APS [3], remains elusive. Renal involvement is a rare manifestation of APS with unfavorable outcomes [4] and inadequate response to standard treatment [5], highlighting a treatment gap.

Herein, we examine whole-transcriptome expression profiles of kidney samples from patients with primary APS *vs* those from systemic lupus erythematosus (SLE) patients, and control individuals.

## Patients and methods

We performed RNA sequencing on archival formalin-fixed paraffin-embedded (FFPE) kidney tissues from primary APS patients with acute and/or chronic APS nephropathy histological lesions, as previously defined [3, 6, 7]. All four patients with biopsy-proven APS nephropathy fulfilled the 2023 EULAR/ACR classification criteria for APS [3]. Patients with APS who concurrently met the classification criteria for SLE [8] were excluded. We also examined kidney tissue samples from two comparison groups: five SLE patients with biopsy-proven lupus nephritis (first diagnosed) and three individuals without any evidence of underlying autoimmune disorder who underwent nephrectomy for renal masses suspicious for malignancy that were eventually diagnosed as oncocytomas; kidney samples from the healthy parenchyma were obtained before formalin fixation, and no abnormal findings were observed on light and immunofluorescence microscopy (controls).

We excluded patients with concurrent autoimmune disorders and those who received high intravenous glucocorticoid treatment or immunosuppressive treatment before the biopsy. The study was approved by the Laiko Hospital Scientific Council (number 859, 25/1/2023), according to the Helsinki Declaration. All patients gave their written informed consent for kidney biopsy. Patients' demographic, clinical and histological characteristics were recorded.

### RNA extraction, sequencing, mapping, quality control and quantifications

All tissue samples proceeded for RNA extraction and sequencing were fully anonymized. Total RNA was extracted from FFPE sections (4–7 sections per sample, section dimensions: 1–3 mm 2× 6 mm) with the FPE RNA/DNA Purification Plus kit (Norgen Biotek) according to the manufacturer’s protocol, using the option of on-column DNase treatment. Isolated total RNAs were quantitated on a Qubit 4 fluorometer using the Qubit RNA HS Assay Kit (Invitrogen/Thermo Fisher Scientific, Whaltham, MA, USA). RNA integrity (RIN, DV200, DV100) was determined on an Agilent 2100 Bioanalyzer, using the Agilent RNA 6000 Nano reagents (Agilent). Selected RNAs (up to 10 ng/sample) were processed into Illumina-compatible libraries using the SMARTer Stranded Total RNA-Seq Kit v3 - Pico Input Mammalian (Takara). The libraries were subsequently quantitated and qualitatively checked on a Qubit 4 fluorometer using the dsDNA HS Assay Kit (Invitrogen) and on an Agilent 2100 Bioanalyzer, using the Agilent High Sensitivity DNA reagents (Agilent). They were then pooled and sequenced on a NextSeq 550 instrument using the Illumina NextSeq 500/550 High Output Kit v2.5, 75 cycles (Illumina).

Following the demultiplexing process, the quality assessment of FASTQ files was conducted using FastQC (version 0.11.9). The raw reads were trimmed for adapters and low-quality bases using Cutadapt (version 1.9.1) through the TrimGalore! wrapper (version 0.6.6). The processed reads were aligned against GRCh38 human reference genome using STAR (version 2.7.6a). Genome tracks for each sample were created and manually inspected. Utilizing GenomicRanges and metaseqR2, the reads were condensed into a reads count table, and normalization was performed using the Bioconductor package DESeq2, as previously described [9]. Genes with less than five reads in >75% of the samples were excluded from the downstream analysis. Dimensionality reduction plot, namely the multidimensional scaling plot, was created for all samples together in R, to project high-dimensional data in 2D.

### Differential gene expression analysis (DGEA) and enrichment analysis

Differential gene expression analysis (DGEA) was conducted on the protein-coding genes using several Bioconductor packages, including DESeq, edgeR, NOISeq, limma and NBPSeq, as previously described [9]. A meta-analysis, facilitated by metaseqR2, was employed to integrate results from multiple algorithms. The PANDORA weighted *P*-value was computed to determine the statistical significance of differentially expressed genes (DEGs). Criteria for identifying DEGs were set at |Fold Change| ≥1.5 and a meta *P*-value <0.05. DGEA was applied for two different comparisons: (i) APS *vs* control kidney samples and (ii) APS *vs* SLE samples.

Enrichment analysis of upregulated and downregulated genes was performed for each of the APS *vs* controls, and APS *vs* SLE comparisons. For the enrichment analysis, the gProfiler R package (version 0.2.2) was used. Enrichment analysis relied on predefined gene sets obtained from protein complexes (CORUM) and pathway databases (Kyoto Encyclopedia of Genes and Genomes-KEGG, Reactome) and the Gene Ontology (GO) terms. The visualization of the results was applied through FLAME [10]. Plots and rest visualization were drawn in R with ggplot2.

## Results

Characteristics of patients with primary APS and those with lupus nephritis are presented in Table 1. All APS patients had APS nephropathy histological lesions (Supplementary Fig. S1, available at *Rheumatology* online); patient #1 had also membranous nephropathy features, as previously reported [6].

**Table 1.** Patient clinical, laboratory and histological characteristics

	Patients with APS				Patients with SLE				
Characteristics	#1	#2	#3	#4	#1	#2	#3	#4	#5
Sex	Female	Male	Male	Male	Female	Male	Female	Female	Female
Age at nephropathy diagnosis	26	51	46	34	44	50	27	37	32
Thrombotic APS^a^ history (months before nephropathy)	Sinus thrombosis (60), DVT (36)	Stroke (48)	DVT (12)	Stroke (1)	No	No	No	No	No
Obstetric APS^a^ history	No	N/A	N/A	N/A	No	N/A	No	No	No
APS microvascular manifestations^a^ (before biopsy)	Skin ulcer	No	No	No	No	No	No	No	No
Heart valve disease^a^	Libman -Sacks	Moderate aortic and mitral regurgitation	No	No	No	No	No	No	No
Thrombocytopenia^a^	mild	moderate	No	No	No	No	Mild	No	No
SLE-related symptoms^b^	No	No	No	No	Yes	Yes	Yes	Yes	Yes
Serum Creatinine (mg/dl)	1.06	1.5	1.2	2.5	0.7	1.1	0.9	1.0	1.5
24h-proteinuria (g)	2.4	2.5	3.0	1.8	0.20	0.30	1.60	3.5	0.35
Hematuria, RBCs/high-power field	3–5	2–3	10–12	8–10	18–20	50–70	15–20	20–25	20–25
Hypertension	160/90	140/90	120/75	180/100	115/70	135/85	125/75	115/65	145/92
Anticardiolipin antibodies	High IgG and IgM titres	High IgG titres	Medium IgG titres	Medium IgG titres	Negative	Negative	Medium IgG titres	Negative	Negative
Anti-β2glycoprotein-I antibodies	High IgG and IgM titres	Medium IgM titres	High IgG titres	Medium IgG titres	Negative	Negative	Negative	Negative	Negative
Lupus anticoagulant	Positive	Positive	Negative	Negative	Negative	Negative	Negative	Negative	Negative
ANA	Negative	Negative	Positive (1/160 sp)	Negative	Positive	Positive	Positive	Positive	Positive
Anti-dsDNA antibodies	Negative	Negative	Negative	Negative	Negative	Positive	Positive	Positive	Negative
Low C3 levels	No	Yes	No	No	Yes	No	Yes	Yes	Yes
Low C4 levels	No	No	No	No	No	Yes	Yes	No	Yes
Treatment prior to biopsy	Warfarin	Warfarin	Warfarin	None	GCs, HCQ, azathioprine	GCs, HCQ,	GCs, MMF	HCQ	GCs, MTX
*Kidney biopsy characteristics*									
Thrombotic microangiopathy (TMA) in glomeruli or arterioles/arteries^d^	+	_	_	Fibrin thrombi in glomeruli and arterioles	_	_	_	_	_
Arterial or arteriolar organized microthrombi with or without recanalisation^d^	–	_	_	+	_	_	_	_	_
Fibrous and fibrocellular [arterial or arteriolar] occlusions^d^	_	_	+	_	_	_	_	_	_
Focal cortical atrophy with or without thyroidization^d^	_	+	_	_	_	_	_	_	_
Fibrous intimal hyperplasia^d^	+	+	+	+	_	_	_	_	_
Ιmmunofluorescence	IgG (3+), IgA: negative, IgM (1+), C1q (1+), C3 (2+), kappa (2+), lambda (2+)	Negative	IgA (1+)	C3 (2+)	IgG (2+), IgA (2+), IgM (2+), C1q (3+), C3 (3+), C4 (3+), kappa (1+), lambda (3+)	IgG (3+), IgA (2+), IgM (2+), C1q (3+), C3 (3+), kappa (2+), lambda (2+)	IgG (3+), IgA (3+), IgM (2+), C1q (3+), C3 (2+), C4 (2+), kappa (2+), lambda (3+)	IgG (2+), IgA (2+), IgM (2+), C1q (2+), C3 (2+), C4 (2+), kappa (2+), lambda (3+)	IgG (3+), IgA (2+), IgM (2+), C1q (3+), C3 (3+), kappa (2+), lambda (3+)
Additional histologic features	Membranous nephropathy. No fibrosis/interstitial inflammation	Moderate interstitial inflammation/fibrosis/(40%)	Mild interstitial inflammation/fibrosis (20%)	Moderate interstitial inflammation/fibrosis (30%)	No fibrosis/interstitial inflammation.	Mild interstitial inflammation/fibrosis (20%)	Mild interstitial inflammation/fibrosis (10-15%)	Moderate interstitial inflammation/fibrosis (25-30%)	Mild interstitial inflammation/fibrosis (20%)
Lupus nephritis classification^e^	_	_	_	_	Class III, AI : 2, CI : 1	Class III, AI : 5, CI : 4	Class V&III, AI : 3, CI : 4	Class III, AI : 6, CI : 7	Class IV, AI : 10, CI : 4

a As defined by the 2023 ACR/EULAR APS classification criteria. Venous thromboembolism events were unprovoked and in the absence of venous thromboembolism risk factors, and the arterial thrombosis was developed in the absence of cardiovascular risk factors.

b SLE-related symptoms: malar rash, discoid rash, serositis, photosensitivity, oral ulcers, arthritis, alopecia, leukopenia, hemolytic anaemia, neuropsychiatric SLE manifestations.

c Thrombocytopenia: Mild: platelet levels between 101 000 and 140 000 per microliter, Moderate: platelet levels between 51 000 and 100 000 per microliter of blood.

d APS nephropathy histologic lesions: as defined in the 2023 ACR/EULAR antiphospholipid syndrome classification criteria: (a) acute renal vascular or glomerular thrombotic microangiopathy lesions, including fibrin thrombi in arterioles or glomeruli without inflammatory cells or immune complexes; and (b) chronic renal vascular or glomerular lesions, described as arterial or arteriolar organized microthrombi with or without recanalisation, fibrous and fibrocellular (arterial or arteriolar) occlusions, focal cortical atrophy with or without thyroidization, fibrous intimal hyperplasia, or chronic/organized glomerular thrombi.

e Lupus nephritis classification according to ISN/RPS (2003/2018 revision).

APS: antiphospholipid syndrome; DVT: deep vein thrombosis; GCs: glucocorticoids; HCQ; hydroxychloroquine; MMF: mycophenolate mofetil, MTX: methotrexate; N/A: not applicable; RBCs: red blood cells; SLE: systemic lupus erythematosus.

**Figure 1.**
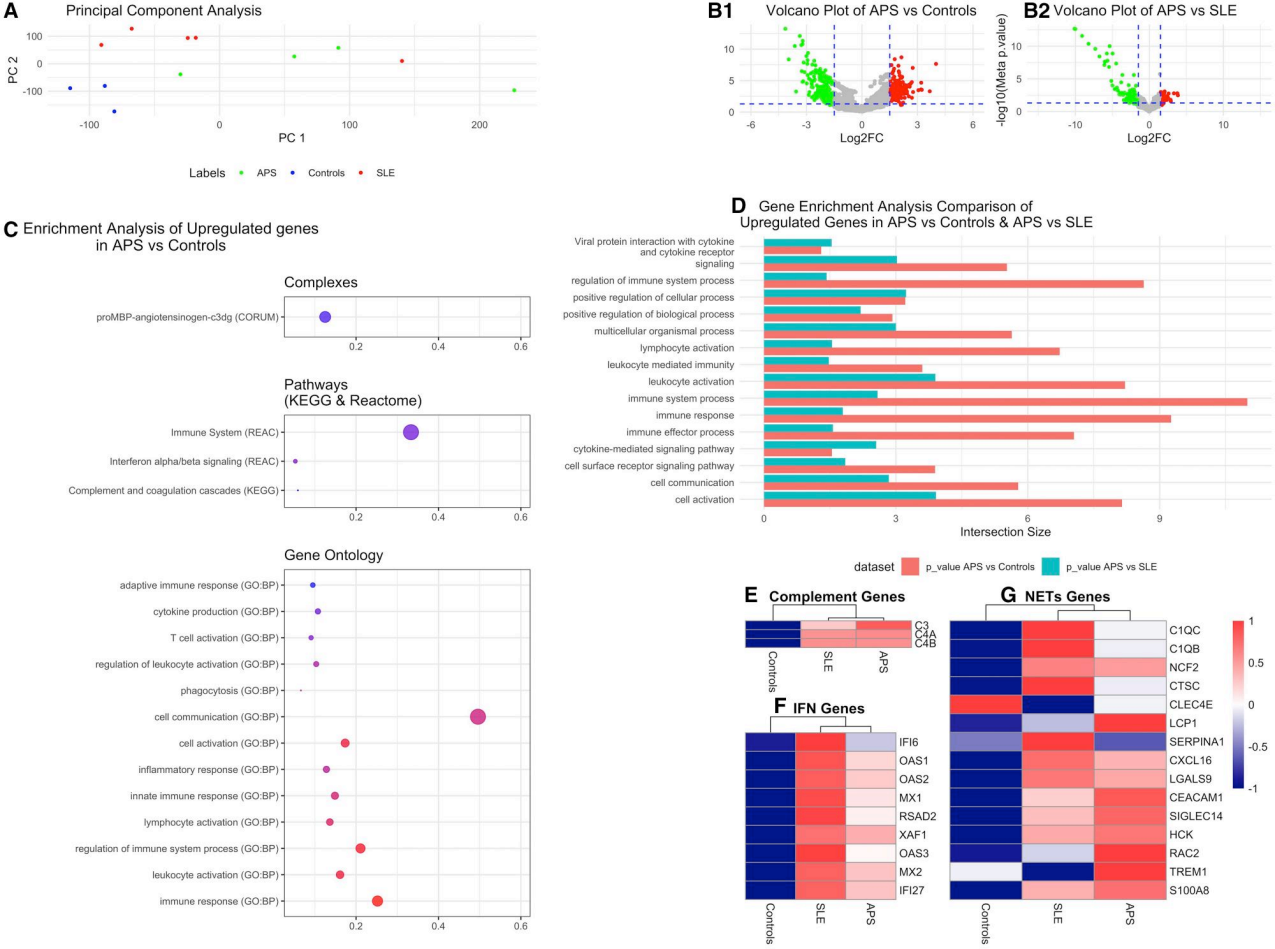
RNA sequencing in kidney tissues of primary antiphospholipid syndrome (APS), systemic lupus erythematosus (SLE) and control individuals. **A**. Dimensionality reduction of all kidney biopsy samples. Principle Component Analysis was applied in order to depict the samples in the 2D space. Each dot corresponds to a sample. The smaller the distance between each sample pair, the greater the similarity of the gene expression profiles. **B**. Illustration of the relationship between statistical significance and magnitude of fold change for each gene in a comparative analysis between APS and control kidneys (**B1**), and between APS and SLE (**B2**). Each point represents an individual gene. The x-axis displays the log2 fold change, indicating the magnitude of the change in gene expression. Positive values signify upregulation, while negative values represent downregulation. The y-axis represents the -log10 of the meta *P*-value, reflecting the statistical significance of the observed changes. Higher values indicate greater significance. The significance threshold is set at meta *P*-value of 0.05 and log2FC >|1.5|, indicating with green colour the downregulated and with red colour the upregulated genes. **C**. Enrichment analysis results for upregulated genes in APS versus control kidney samples. Selected terms are presented. The x-axis represents the enriched terms, the y-axis shows the precision, the size of the points indicates the intersection size, and the colour corresponds to the -log10 of the *P*-value of enrichment (red and blue colour indicates higher and lower significance, respectively). **D**. Comparison of enriched terms in APS versus controls and APS versus SLE kidney samples. The bar plots illustrate the intersection of enriched terms among the upregulated genes in the comparisons between APS versus controls and APS versus SLE. In the color scheme, red bars represent the APS versus controls comparison, and the blue bars represent the APS versus SLE comparison. The y-axis denotes the enriched terms, while the x-axis represents the number of genes intersecting with each term. **E**. Complement-related genes expression in APS, SLE and control kidney samples. Heatmap representation that displays the z-score-scaled mean normalized expression values of the three complement-related genes (C3, C4A, C4B) found up-regulated in APS samples in the differential expression analysis. **F**. Interferon-regulated genes expression in APS, SLE and control kidney samples. Heatmap representation that displays the z-score-scaled mean normalized expression values of the nine interferon-regulated genes found upregulated in APS samples in the differential expression analysis. **G**. NETs-related gene expression in APS, SLE and control kidney samples. Heatmap representation that displays the z-score-scaled mean normalized expression values of the 15 neutrophil extracellular traps (NETs)-related genes that were found to be expressed in kidney specimens of patients with APS

Dimensionality reduction plot projection in 2D space revealed a clear separation between APS and controls but not between APS and SLE patient samples (Fig. 1A). DGEA of APS *vs* controls revealed 276 upregulated and 217 downregulated genes (Supplementary Table S1, available at *Rheumatology* online), while a comparison of APS *vs* SLE identified 75 upregulated and 111 downregulated genes (Supplementary Table S2, available at *Rheumatology* online). Volcano plots highlight more pronounced differences in kidney transcripts between APS and controls than between APS and SLE samples (Fig. 1B).

Enrichment analysis of 276 upregulated genes in APS *vs* control kidneys is presented in Supplementary Table S3, available at *Rheumatology* online. Upregulated genes were mainly enriched in GO terms of (innate) immune response, inflammatory response, leucocyte and lymphocyte activation, cytokine production and T-cell activation (Fig. 1C). Although these terms were common in both APS *vs* controls and APS *vs* SLE comparisons, a higher number of enriched genes was observed in the former comparison (Fig. 1D).

CORUM and KEGG enrichment analysis revealed complexes and pathways related to the complement cascade (Fig. 1C). In this term, we further examined the expression values of the three complement-related genes (C3, C4A and C4B) found upregulated in APS compared with control samples (Fig. 1E). Indeed, differential expression analysis comparing APS *vs* controls showed logFC values of 2.25 (*P* = 1.58e-05) for C3, 2.17 (*P* = 2.69e-06) for C4A, and 2.135 (*P* = 3.7e-06) for C4B (Supplementary Table S1). Conversely, we found comparable expression levels between APS and SLE samples. No significant difference was observed in complement regulatory genes’ expression between APS and controls.

One of the most enriched terms in the Reactome database was ‘Interferon (IFN) alpha/beta signalling’ (Fig. 1C). Thus, we further examined the expression values of the nine type I/II IFN-regulated genes found to be upregulated in APS compared with controls (Fig. 1F). Interestingly, differential expression analysis in APS *vs* controls showed *P*-values ≤0.001 for all nine IFN-regulated genes (Supplementary Table S2). Although higher logFCs were observed in SLE than in APS for all IFN-regulated genes, no significant difference was reached (Supplementary Table S1).

Based on the potential role of neutrophil-extracellular traps (NETs) in APS [1, 11], we compared the expression of 16 NETs-related genes [12] in kidney samples of APS *vs* controls and SLE. One of the above genes was not expressed in our dataset. Remarkably, 13 of 15 NETs-related genes exhibited higher expression in APS *vs* controls samples (Fig. 1G). SERPINA1 and CLEC4E genes had the highest expression in SLE and control kidney samples, respectively.

## Discussion

To our knowledge, this is the first RNA-sequencing study in kidney biopsy samples from patients with primary APS. We observed higher expression of complement, IFN and NETs-related genes in primary APS *vs* control kidney tissues, and similar to that detected in lupus nephritis samples.

APS is a complex autoimmune disorder characterized by a plethora of obstetric, thrombotic and microvascular manifestations in association with persistently positive aPL. Despite significant advances over the past four decades, the pathogenesis of APS, especially of its less well-characterized or less frequent clinical features like APS nephropathy, remains poorly defined. Better identification of disease pathophysiology, as well as molecular pathways and biomarkers associated with various clinical phenotypes, will facilitate a more personalized treatment approach. Recent research supported the role of antiphospholipid antibody-mediated inflammatory mechanisms on endothelial activation and injury in APS and their interplay with the coagulation cascade pathways. Innate immunity responses are increasingly recognized in APS, involving monocytes, cytokines and chemokines expression, as well as complement, platelets and tissue factor-enriched neutrophils activation, opening the discussions about targeted treatments in APS [1, 2, 11].

In the APS kidney samples, we found highly expressed complement C3, and C4A and C4B genes which encode the basic form of complement factor 4, all parts of the classical complement pathway. Earlier mechanistic studies demonstrated the role of complement activation in obstetric APS, and more recently, in thrombotic APS as well [13]. New aspects, such as complement system interrelationship with platelets and NETs are gaining attention [11, 13], along with its role in thrombotic microangiopathy complications in APS, including the catastrophic APS [13], and potentially in APS nephropathy based on data from an animal model [14]. Complement inhibition treatment has been approved in complement-mediated thrombotic microangiopathy syndromes such as atypical hemolytic syndrome. It has also been successfully introduced in catastrophic APS cases, with some promising data in patients with lupus nephritis and thrombotic microangiopathy [15], suggesting its potential use also in APS nephropathy.

We also found higher logFCs for type I and II IFN-regulated genes in APS kidney FFPEs *vs* controls, and similar to SLE samples. Recent studies showed a prominent type I IFN signature in PBMCs or whole blood from primary APS patients and similar or slightly lower expression compared with that observed in SLE and SLE/APS patients [9, 16, 17]. The IFN signature promoted impaired endothelial progenitor function which was successfully reversed by a type I IFN receptor-neutralizing antibody [18], underlying the need for a further investigation of IFN inhibition to prevent vascular damage in APS.

The role of neutrophils and NETs in thrombosis, the link between C5a and neutrophil activation in pregnancy complications, and their interferogenic role have been acknowledged in APS and SLE [19]. Recently, mRNA expression of NETs-associated genes (MPO, ELANE and PADI4) was described in leukocytes from patients with thrombotic primary APS, especially those with recurrent thrombosis [20]. However, it remained unclear whether NETs are involved in APS manifestations other than thrombosis, such as microvascular features, and how their antagonism might affect these specific disease phenotypes. We found, for the first time, higher NETs-related gene expression in kidney tissues from patients with APS *vs* controls.

Notably, in a previous whole-blood RNA-sequencing study of 62 patients with thrombotic APS *vs* 29 healthy controls, we found similar complement (C4A, C4B) and type I/II IFN-regulated genes expression to that detected in the present kidney transcriptome study [9]. Deconvolution analysis also revealed upregulated genes of neutrophils among primary APS patients with history of arterial thrombosis.

Upregulation of complement, IFN and NETs-related genes has also been demonstrated by some studies examining kidney biopsy transcriptomes or whole-blood transcriptomic profiles in patients with lupus nephritis [15, 21–23]. Primary APS and SLE, although distinct entities [3], share several susceptibility genes [2] and clinical and serological characteristics: antiphospholipid antibodies (aPL) occur in 30–40% of SLE patients; antinuclear antibodies, Coombs-positivity and low complement levels are present in some primary APS patients; and APS nephropathy co-exists with lupus nephritis lesions in kidney biopsies from SLE/aPL-positive patients [7]. None of our APS nephropathy patients met SLE criteria [8], and all had an extended follow-up (4–12 years) excluding a transition from PAPS to SLE.

APS nephropathy is a rare manifestation with a poor renal prognosis, that is often overlooked in primary APS. This is due to its rarity, underestimation of its clinical presentation, and reluctance to perform kidney biopsies in these patients because of the anticoagulation treatment used by most patients or concurrent thrombocytopenia [4]. Thus, renal biopsy samples in primary APS are rarely available, explaining the size of our sample and the inclusion of archived FFPE biopsy specimens.

Our kidney tissue transcriptome data highlight potential treatment targets in APS [24] such as complement or IFN inhibitors, or NETs-targeted therapies. Confirmation of our findings in multicentre studies is warranted.

## Supplementary Material

keae397_Supplementary_Data

## Data Availability

Data will be made available upon request.
