# Supplementary material for: Kidney whole-transcriptome profiling in primary antiphospholipid syndrome reveals complement, interferons and NETs-related gene expression
Source: Rheumatology (Oxford). 2024 Aug 6;63(11):3184–90. doi: 10.1093/rheumatology/keae397 (PMC11534096; doi:10.1093/rheumatology/keae397)
Supplement: keae397_Supplementary_Data [file keae397_supplementary_data.docx]

**Supplementary Figure S1. Kidney biopsy lesions in patients with primary APS.**

A. Fibrin thrombus (arrow) in an arteriole (patient 4) / Masson trichrome x 200; B. Fibrin thrombi (arrow) in glomerular capillaries (patient 4) / H&E x 400; C. Fibrous intimal hyperplasia (arrow) in a small interlobular artery (patient 2) / Masson trichrome x 400; D. Prominent circumferential intimal cellular proliferation (arrow) in an arteriole (patient 3) / H&E x 600

**
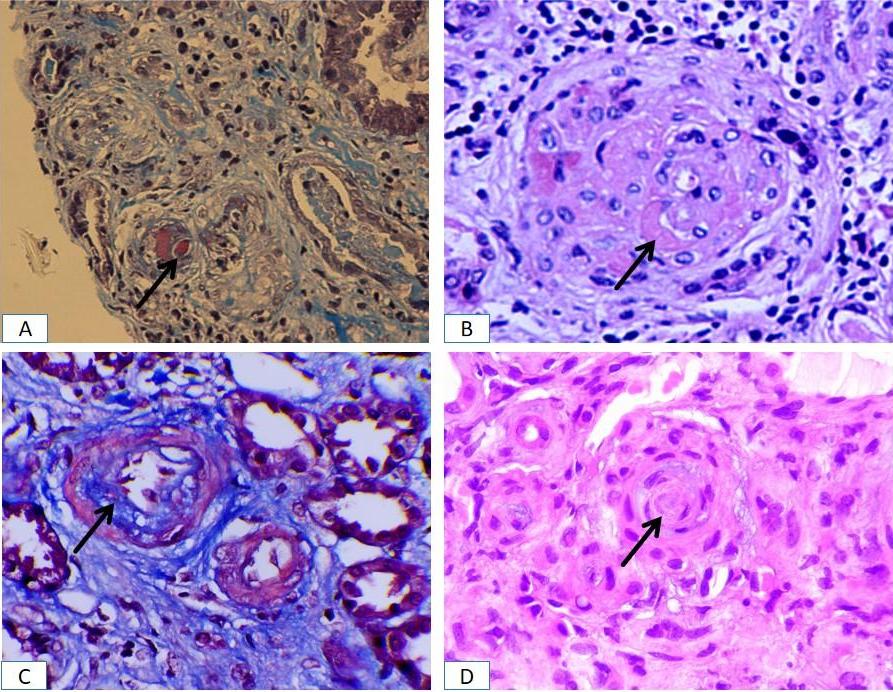
**

**Supplementary Table S1.** Upregulated and downregulated genes for the comparison between patients with APS and healthy controls

| **Upregulated APS vs HC** |  |  |
| --- | --- | --- |
| **gene_name** | **meta_p-value_APS_vs_HC** | **log2_normalized_fold_change_APS_vs_HC** |
| DUX4 | 2.20251089901819e-08 | 4.00030459095244 |
| GFAP | 0.000496977403066454 | 3.66177809777199 |
| ADGRG7 | 0.000288908923942873 | 3.23120134180687 |
| IFI44L | 2.15008649640588e-05 | 3.20038041603629 |
| APOB | 0.000253754800838835 | 3.19140472885276 |
| SYNDIG1L | 0.000333748897949761 | 3.04890960048095 |
| IFI6 | 0.000652503592641787 | 2.76055859786501 |
| NKAIN4 | 9.61769759494488e-08 | 2.74101532758191 |
| FGB | 0.00530616482120214 | 2.69647081593815 |
| OTOF | 1.73052073634599e-05 | 2.69348695749933 |
| OAS1 | 3.03289778483284e-05 | 2.62875098007202 |
| CMPK2 | 9.15880682327344e-05 | 2.57760447727696 |
| SLC1A7 | 8.37774640363551e-05 | 2.57102330976328 |
| OAS2 | 4.4998683213935e-05 | 2.55014914214009 |
| C10orf71 | 0.00316691119537212 | 2.53092266100827 |
| FCRL5 | 0.000549519461195495 | 2.43092887204874 |
| PLPPR5 | 0.00107896722634474 | 2.43045255166553 |
| TLR7 | 0.000443602641874344 | 2.40987579416307 |
| SLAMF7 | 5.99732978584275e-05 | 2.40636519726439 |
| CCL15 | 7.26563885914766e-05 | 2.40209844357135 |
| MX1 | 0.000124907170478103 | 2.39283843941169 |
| C16orf54 | 0.000163027538247475 | 2.38370429247405 |
| GAGE12F | 0.000686989213237132 | 2.37155886261196 |
| CYP21A2 | 0.000268359319550421 | 2.34129341975429 |
| CCL19 | 0.00537380645212212 | 2.30624647265509 |
| GOLGA6L2 | 0.000513919483506922 | 2.30237529746596 |
| MUC13 | 4.92718264828539e-05 | 2.29797871853756 |
| ZBP1 | 0.001205578772111 | 2.28095631383106 |
| ACKR1 | 0.0317775939120014 | 2.22881869049588 |
| SIGLEC1 | 0.00130566121574037 | 2.21939367770661 |
| MUC12 | 1.75678027236246e-05 | 2.20767992343773 |
| RSAD2 | 0.00125966716013426 | 2.18927241564511 |
| XAF1 | 0.000114913728559609 | 2.1782899006252 |
| STRIP2 | 2.56698999004317e-06 | 2.17136841831198 |
| C4A | 2.69326329942302e-06 | 2.17030504808208 |
| C4B | 3.69582599425293e-06 | 2.13483905683851 |
| LILRA1 | 0.00102633675141031 | 2.13245029602365 |
| GDA | 1.99871764675687e-09 | 2.13156762109366 |
| MUC17 | 0.00576566279461223 | 2.12653240592893 |
| PRF1 | 0.000252237695950592 | 2.12412131182919 |
| RUFY4 | 0.00064318594505723 | 2.11869967808714 |
| CYTH4 | 3.41678354730347e-06 | 2.10202093694051 |
| PGA5 | 0.0394906491476876 | 2.08246216019197 |
| ANXA13 | 3.41187899254826e-05 | 2.07998287612951 |
| DYTN | 0.000656235185467247 | 2.07850197367872 |
| DRD2 | 0.00234372207851529 | 2.0753963199655 |
| TMEM132E | 3.45296012537126e-05 | 2.07006625207751 |
| APOC1 | 0.00089100389884589 | 2.04439411935845 |
| HMOX1 | 1.6608296550034e-06 | 2.03993293890344 |
| SYT6 | 1.51785438365823e-06 | 2.03638865665607 |
| VSTM2B | 0.00115011050885371 | 2.03426990208475 |
| IFI44 | 0.00053598203697949 | 2.03309547678938 |
| GAGE1 | 0.00223302470681796 | 2.03242147769238 |
| OAS3 | 0.000397050926016154 | 2.02558612031621 |
| C3 | 1.58392121339725e-05 | 2.02474127720619 |
| GOLGA6L22 | 2.97066470875975e-05 | 2.02345897282399 |
| COL17A1 | 0.00235198597950425 | 2.02160225448521 |
| F12 | 0.00202257577533904 | 2.01792190799726 |
| ENPP7 | 8.53962387812781e-05 | 2.01559685505102 |
| IL2RB | 4.76263870093019e-05 | 2.01158797427521 |
| SLC34A1 | 5.28550542743313e-07 | 2.00848976381732 |
| MX2 | 0.000592280895692915 | 2.00468608084057 |
| SLC17A3 | 3.37487173228123e-07 | 1.98842552739602 |
| ARTN | 0.00577953923546333 | 1.9870609442925 |
| CCDC194 | 0.00148943013028613 | 1.98413359454098 |
| MEGF11 | 9.21142684042018e-06 | 1.98360961084564 |
| ABCB11 | 0.00100241650147213 | 1.97949434456536 |
| RNF183 | 0.000331531009768495 | 1.97887670843425 |
| EPSTI1 | 0.000337464418301497 | 1.9720206768594 |
| ZAP70 | 2.85339211061833e-05 | 1.9719856238304 |
| CDH6 | 4.27169238161242e-08 | 1.97086508393649 |
| CEACAM5 | 0.0193988750761493 | 1.96962635095648 |
| TIFAB | 0.00463852739680098 | 1.96347412397489 |
| ODF3B | 0.000376209311406385 | 1.95256817000316 |
| RAC2 | 2.17872567629506e-05 | 1.94840986504679 |
| FCAMR | 1.89316876726974e-05 | 1.94462200625428 |
| PLPP2 | 0.000459109333943195 | 1.92444896699282 |
| KCNA2 | 0.000209198904759028 | 1.92226582149543 |
| IFI27 | 0.00049784695735454 | 1.92004070141768 |
| SOST | 0.00426356312968578 | 1.91986289275427 |
| MZB1 | 0.0128453351151113 | 1.91838623444635 |
| LHX6 | 8.39499198659634e-05 | 1.91436571617067 |
| STMN2 | 0.00342528234216788 | 1.90689059560852 |
| CYP26C1 | 0.00220096102929441 | 1.88752527074159 |
| SLC22A12 | 4.47128090276897e-05 | 1.87453023222962 |
| KLHDC7B | 0.00242395061721067 | 1.87184364850932 |
| OOSP3 | 0.000274783665607821 | 1.86507041991389 |
| SLC30A8 | 3.60600912692004e-05 | 1.86453658270488 |
| CD52 | 0.0014528388477364 | 1.86108690599539 |
| SPIB | 0.00129135518105047 | 1.85958488083016 |
| CEBPA | 0.000152951852836409 | 1.85879630740747 |
| TNFSF14 | 0.00352033131744205 | 1.85798099512757 |
| TRIM71 | 1.72851094682961e-05 | 1.85395846479367 |
| PNLIPRP3 | 0.00159501705102835 | 1.85345133664706 |
| MKI67 | 0.000263221773832791 | 1.84940510094776 |
| OPN5 | 0.00480886132484357 | 1.84482962747627 |
| MYO1A | 0.00529770721947198 | 1.84482962747627 |
| AKR1B10 | 0.00864996412950042 | 1.8427602581888 |
| SLC5A2 | 2.08562263995743e-05 | 1.84059370857325 |
| SLC6A11 | 0.00167658447099149 | 1.84021955596323 |
| B3GNT4 | 0.0013231970788781 | 1.83977639974998 |
| HERC6 | 0.00183708024938386 | 1.8376279331714 |
| TNNT3 | 0.0127570549175808 | 1.83289001416474 |
| TRIM6 | 9.90921255611047e-05 | 1.83270158667921 |
| NYAP2 | 0.000106090092855067 | 1.83232745028907 |
| SLC51B | 0.000425410738264364 | 1.83127354896382 |
| TMC3 | 0.00119782564272123 | 1.83043853517065 |
| WDFY4 | 9.94748625456113e-06 | 1.82572080452856 |
| CNPY1 | 0.0019423860624769 | 1.82501358855411 |
| TBX5 | 0.00768504729947707 | 1.82442843541655 |
| KCNA3 | 0.00606846277588826 | 1.82200169802201 |
| LHX2 | 0.00358420869261214 | 1.81942775435818 |
| KEL | 0.000970404340756891 | 1.81845263094093 |
| IL21R | 3.44330847404589e-05 | 1.81690922877085 |
| GOLGA8F | 0.0013244599600798 | 1.811030580201 |
| COMP | 0.0038434325245304 | 1.80952275874031 |
| CBLN3 | 0.000341658382563766 | 1.80913054694746 |
| FCGR3A | 0.000605869036564483 | 1.7987417917529 |
| C14orf180 | 0.00122120981544454 | 1.79795622405535 |
| NOD2 | 0.000203352882569478 | 1.7968815908281 |
| IQSEC3 | 1.10481031140777e-05 | 1.79550461067835 |
| DPEP2 | 0.000141124458268675 | 1.7951802081115 |
| PLA2G7 | 0.00238957709587289 | 1.78805436609867 |
| GMNC | 0.000433185021389707 | 1.78290187833307 |
| GOLGA6L1 | 9.8444444070882e-05 | 1.77617106053143 |
| ASPG | 3.65118038893225e-05 | 1.77342706272503 |
| SLC22A13 | 0.00011122823710024 | 1.77147453113525 |
| HABP2 | 0.000553777113038204 | 1.76888077424297 |
| NEK6 | 3.93093760839385e-09 | 1.76856607429383 |
| NRARP | 0.00127598633032804 | 1.76716583194191 |
| GATA4 | 0.00441388354905996 | 1.76428620016572 |
| CPA4 | 0.00577337927417008 | 1.76250068627334 |
| CCL5 | 0.00132448316993843 | 1.76209018832182 |
| OTOGL | 0.000118067315884087 | 1.75425873987812 |
| METTL7B | 0.000133727211899175 | 1.75399169645888 |
| OLFM4 | 0.00483843062560674 | 1.75087443965684 |
| NCF1 | 0.000346620500483547 | 1.75002174699165 |
| IGLL5 | 0.00210728970929079 | 1.74916973375333 |
| CCDC168 | 0.00202119717141615 | 1.74846123300404 |
| BHMT | 0.000285884637462364 | 1.74764241086882 |
| GPR183 | 0.00368349056482303 | 1.74636594731762 |
| ZNF296 | 0.002879351121764 | 1.7454271729144 |
| DPYS | 2.37179436086677e-06 | 1.74524612639636 |
| RELL2 | 0.000740615621383772 | 1.74108170263844 |
| SCEL | 0.000850081882508413 | 1.74024072619907 |
| FMO1 | 6.99315858512667e-05 | 1.73872225953864 |
| MYH15 | 0.00105821564673759 | 1.73382588663564 |
| TINAG | 1.29641540660842e-07 | 1.73345477536906 |
| CYP24A1 | 6.21476640934638e-05 | 1.73141077342062 |
| RNF212B | 0.00108320382140563 | 1.72954812260372 |
| STEAP1 | 0.000363454829052057 | 1.72879827329481 |
| SLC22A7 | 5.09913687144498e-05 | 1.7275123783796 |
| CD48 | 0.000579419231053967 | 1.7219101797172 |
| PLEK | 0.000119741036702865 | 1.71683415639176 |
| CMKLR1 | 4.59111400439167e-06 | 1.71049338280502 |
| RIMS1 | 0.000106742410002622 | 1.70987444703808 |
| PSORS1C1 | 0.000462024615897219 | 1.70872428995196 |
| NXNL2 | 0.000185347792009353 | 1.7079621674271 |
| DRICH1 | 3.43143691646824e-05 | 1.70590353787534 |
| SNX20 | 0.00241474094502847 | 1.70043971814109 |
| TSPAN18 | 2.89062807723909e-07 | 1.69969886137667 |
| RHBDL1 | 0.003134483459942 | 1.69647081593815 |
| LGALS9B | 0.00287061594563721 | 1.69538649041481 |
| SLC22A14 | 4.04341290358578e-05 | 1.69479315499995 |
| KIF19 | 0.00158262930177291 | 1.69075716474375 |
| CHAT | 0.00331302800591248 | 1.69075716474375 |
| MROH2A | 0.000419077468919625 | 1.6902824065978 |
| GOLGA6L6 | 0.000172787869847008 | 1.68929916053589 |
| CLRN3 | 0.00010395244411652 | 1.68851869254775 |
| GNLY | 0.00259638623181795 | 1.68604862583547 |
| SLC22A18AS | 5.07635115166471e-05 | 1.68362881899365 |
| ADM2 | 0.000155566510063088 | 1.68316911379955 |
| LPAR3 | 0.00252647849771076 | 1.68088692071969 |
| SIRPG | 0.0020187371231351 | 1.68072148352659 |
| ITGAL | 3.72232554512592e-05 | 1.68045849794952 |
| ABCC12 | 0.00959748637818892 | 1.6801197337615 |
| CBLIF | 0.0111547514607547 | 1.6801197337615 |
| SMLR1 | 0.00277367904774825 | 1.6786216079354 |
| CRHBP | 0.000175628714798849 | 1.67641032298105 |
| SLC6A19 | 0.000223173828741042 | 1.67255609785511 |
| WNT3A | 0.0040132682636144 | 1.6724253419715 |
| CD180 | 0.00467454099001668 | 1.66588249610472 |
| BCAN | 0.00688223683019398 | 1.66418919187173 |
| EHD3 | 0.000390440944064988 | 1.65628276343982 |
| ANPEP | 4.65567013305188e-06 | 1.65583454446232 |
| DERL3 | 0.0026994677940579 | 1.65313400336274 |
| SLC13A5 | 0.00330216897470654 | 1.64797229824696 |
| LRP2 | 5.57343904191789e-07 | 1.64649969839964 |
| TMEM212 | 0.00530918176854507 | 1.64550404265621 |
| GLP1R | 0.00631551819996002 | 1.64466874684542 |
| EVI2B | 0.00130626112346327 | 1.64167689171548 |
| DHH | 0.00685646009100859 | 1.64154602908752 |
| PAH | 7.01098585815904e-07 | 1.64111824890467 |
| HPD | 6.50534673635908e-05 | 1.64042785900415 |
| ERICH3 | 0.00164384861221365 | 1.63662462054365 |
| UBASH3A | 0.000301251267987005 | 1.63396984145761 |
| SASH3 | 0.00048565510157293 | 1.6338721012021 |
| ACAN | 6.95858302321117e-05 | 1.62800602512038 |
| SYT16 | 0.000113669091258153 | 1.62744348488228 |
| CAV3 | 0.00626610659060321 | 1.62630329610206 |
| OR2T12 | 0.0034030080585877 | 1.62449086490779 |
| TRAPPC3L | 0.00758831292351763 | 1.62309762960793 |
| FAM163A | 0.00585384985008847 | 1.62216111867541 |
| PLA2G5 | 0.00241102025010489 | 1.62148837674627 |
| LAMP5 | 0.00965747155096762 | 1.62058641045188 |
| SOX18 | 6.96010491827276e-05 | 1.61649807415426 |
| SMCO2 | 0.00334238426638586 | 1.61470984411521 |
| SLFN12L | 0.00251168715991272 | 1.61306783840151 |
| CD33 | 0.00315326002306688 | 1.61297687689075 |
| IL22RA1 | 0.000236540326356725 | 1.60253513016911 |
| APLNR | 0.0172748967394308 | 1.60169651648096 |
| A1CF | 7.29423420550136e-05 | 1.60130318556406 |
| PRR5 | 3.39203242328947e-08 | 1.5956582143169 |
| STC2 | 0.000398038080317332 | 1.59503957340911 |
| OTOG | 0.00138504496657227 | 1.59245703726808 |
| AGT | 0.000355892508822683 | 1.59020549270921 |
| NLRC3 | 6.47981190754326e-05 | 1.58897928765255 |
| P2RY6 | 2.65999557639556e-05 | 1.58896443127865 |
| FAM151A | 0.000333659475915785 | 1.58578855000848 |
| NPFFR2 | 0.00126492499303429 | 1.58496250072116 |
| AL590764.2 | 0.00182942089795253 | 1.58496250072116 |
| OR4N2 | 0.00330598395981523 | 1.58496250072116 |
| ERICH6B | 0.00742003484962548 | 1.58496250072116 |
| CEMP1 | 0.0112930755106463 | 1.58496250072116 |
| PDCD1 | 0.0140378020966136 | 1.58496250072116 |
| SIGLEC6 | 0.0161613594457941 | 1.58496250072116 |
| AC067752.1 | 0.000196810536366479 | 1.58244690280237 |
| VAV1 | 0.000144305340430897 | 1.57672492555091 |
| GJB2 | 0.00125060810667339 | 1.57399138290015 |
| FCN3 | 0.00110471502143523 | 1.57288966842058 |
| IL2RG | 0.00294127473673449 | 1.57268417094272 |
| GPA33 | 0.0024808439649459 | 1.57178711197342 |
| CD244 | 0.00179168212137479 | 1.57046293102604 |
| GGTLC2 | 0.000169419635830715 | 1.56644241275036 |
| ADCY7 | 6.72915062427639e-05 | 1.566197818444 |
| E2F8 | 0.0102802368344688 | 1.56478461878353 |
| ITGB2 | 0.000224032748983077 | 1.56447902779723 |
| CD68 | 3.31492346947675e-05 | 1.56368970243061 |
| GATA1 | 0.00557066405555481 | 1.56358884992859 |
| CHST13 | 0.00056259182095902 | 1.55987151975833 |
| CYP26B1 | 0.0088914227798337 | 1.55942740861402 |
| SPACA3 | 0.0162695319788048 | 1.55896729218821 |
| LGALS2 | 0.000124206085532948 | 1.55774103198613 |
| RELT | 5.4441098985259e-05 | 1.55563383545557 |
| PARP15 | 0.000712578837457497 | 1.55557869394006 |
| LMX1A | 0.00767876630459133 | 1.55254102302878 |
| ST8SIA4 | 0.000119715346248095 | 1.54873253179192 |
| TYMP | 0.00065653882463827 | 1.54834617643164 |
| TMEM74 | 0.00459823157138414 | 1.54780950230369 |
| NETO1 | 0.00142631073826924 | 1.54365552656424 |
| ARL4C | 0.00122262981808338 | 1.54273426533186 |
| MYO1G | 0.000228513047225382 | 1.54206454228344 |
| ADRA1D | 0.00262160405175479 | 1.53915881110803 |
| IGSF11 | 8.82622412951458e-05 | 1.53873948396051 |
| DTX4 | 1.0662557358226e-05 | 1.53685659058246 |
| PSTPIP1 | 0.000416388807587989 | 1.53684055110298 |
| PAX5 | 0.000267160437639479 | 1.53138146051631 |
| FUT3 | 8.42237741713838e-05 | 1.52711842614887 |
| GGTLC3 | 0.000703037815827479 | 1.52699490244993 |
| SLC17A1 | 0.000692896802591241 | 1.52638892307695 |
| PSAT1 | 0.00011020530825345 | 1.52347397880047 |
| SELPLG | 0.000389309049523474 | 1.52127921026289 |
| LYL1 | 0.000611777889901726 | 1.52105073690096 |
| TMC8 | 0.000463799510215439 | 1.5120309782673 |
| AIFM3 | 0.00103613284194217 | 1.50923517455791 |
| LY6E | 0.00091643388534732 | 1.50732365862613 |
| SLC47A2 | 0.000307487138970074 | 1.50728583541449 |
| LRRC74A | 0.00367322075231445 | 1.50695998871988 |
| TFAP2D | 0.0103913548988281 | 1.50695998871988 |
| TCF7 | 0.000294571260288815 | 1.50587475157518 |
| PTTG1 | 0.014653983526879 | 1.50552803322675 |
| PPM1N | 0.0113073643041311 | 1.50499419601774 |
| CD300A | 0.000546115125732011 | 1.50250034052918 |
| CR2 | 0.00115259804114844 | 1.50250034052918 |
| RXRG | 0.00927755644912068 | 1.50057031343078 |
| RUNDC3A | 0.00259846959987729 | 1.50007360313464 |

| **Downregulated APS vs HC** |  |  |
| --- | --- | --- |
| **gene_name** | **meta_p-value_APS_vs_HC** | **log2_normalized_fold_change_APS_vs_HC** |
| **ARG2** | 5.67244145166363e-14 | -4.13988617506093 |
| **EDN2** | 4.16717921546537e-09 | -3.9584208962486 |
| **EGF** | 3.22399071083003e-11 | -3.63326519610503 |
| **MTRNR2L5** | 0.000548462302104308 | -3.56187888760812 |
| **NR4A1** | 8.06136069834475e-13 | -3.36059174392829 |
| **HOMER1** | 2.2611301350743e-11 | -3.28170571660044 |
| **DDIT4L** | 3.17039807110242e-07 | -3.2545728270856 |
| **SH3GL3** | 1.55700187139229e-05 | -3.21512848690384 |
| **NR4A3** | 1.5843690715497e-11 | -3.21283865081359 |
| **PDK4** | 4.7175750098059e-12 | -3.20489861179045 |
| **NEFL** | 1.27729860457302e-05 | -3.17786126136608 |
| **SLC4A11** | 1.30232773619901e-08 | -3.15279556533583 |
| **GP2** | 4.54929006838599e-07 | -3.10499537976254 |
| **COL25A1** | 1.27158386194536e-09 | -3.00311200149097 |
| **SLC14A2** | 2.82805494950309e-05 | -2.98451978763336 |
| **PLEKHG4B** | 2.20365863228797e-08 | -2.95109039951905 |
| **HSPA2** | 5.65809281775292e-09 | -2.94085088654954 |
| **ARC** | 1.36265437561064e-06 | -2.93859945533586 |
| **TIPARP** | 4.09423131595783e-10 | -2.91656563399412 |
| **TFAP2C** | 0.00190572253077912 | -2.87931851757754 |
| **PADI2** | 4.87756902714916e-06 | -2.85968475241934 |
| **CNTD2** | 0.000668088691438077 | -2.84434912953981 |
| **PROX1** | 8.1048726517951e-06 | -2.76405486928398 |
| **TMEM207** | 6.92107408791095e-07 | -2.76192301698777 |
| **IGF2** | 2.99094824215006e-05 | -2.72539483079044 |
| **UMOD** | 1.26244021358319e-05 | -2.72458278376524 |
| **HJV** | 0.000106477897816235 | -2.71569697741255 |
| **AC132217.2** | 3.49613448181447e-05 | -2.70846383636798 |
| **TIMP4** | 0.00457240147221744 | -2.70626879694329 |
| **RASD1** | 2.83354620704655e-08 | -2.68397931253625 |
| **EGR2** | 0.000116944318416446 | -2.66041472915062 |
| **INS-IGF2** | 4.28988673834468e-07 | -2.64591185068549 |
| **LRRC52** | 0.00291958523212684 | -2.61470984411521 |
| **DACH2** | 2.03552514345372e-06 | -2.59753173693301 |
| **TENT5B** | 1.03568119776456e-05 | -2.5902959450242 |
| **SERPINE1** | 6.97983393844526e-09 | -2.58226005882716 |
| **MTRNR2L4** | 0.00703147376838444 | -2.58181594540429 |
| **SPTSSB** | 0.0124188655262266 | -2.56514105980841 |
| **MMP19** | 6.77737694153986e-06 | -2.54885047731754 |
| **SLC26A3** | 3.74248407408558e-08 | -2.51534240507453 |
| **MELTF** | 6.0642878011596e-07 | -2.51475671014199 |
| **UNCX** | 0.00103911911467292 | -2.51096191927738 |
| **CCN1** | 1.99989332394006e-07 | -2.50381140481506 |
| **SLC22A16** | 0.00307055937824795 | -2.47902559415066 |
| **ANKRD30BL** | 0.0030789215569578 | -2.47345236616425 |
| **GCNT3** | 0.000793393828240408 | -2.4427619822238 |
| **MUC15** | 5.64500122642774e-05 | -2.43949054300338 |
| **RDH8** | 0.00566321583563708 | -2.43673257037816 |
| **LPL** | 3.93535150382301e-07 | -2.40896298486916 |
| **PDLIM3** | 0.000273028640227179 | -2.38599782632749 |
| **ABCA4** | 0.000292867230792178 | -2.38134321403689 |
| **GPM6B** | 3.22731168213063e-07 | -2.37802503907598 |
| **CRISPLD2** | 2.88644580217945e-07 | -2.36972475419708 |
| **SYT1** | 0.000117405387841101 | -2.36432667894531 |
| **OLFM1** | 0.00190640764162171 | -2.34288771352301 |
| **MTRNR2L10** | 0.00252723144592612 | -2.31609564958521 |
| **FILIP1L** | 8.26154573564593e-07 | -2.3125183549692 |
| **RP1** | 2.70669439948257e-06 | -2.29506232991282 |
| **ZFAND5** | 2.60032687659552e-07 | -2.29454773712788 |
| **EGR1** | 6.42890030142275e-07 | -2.28024946618215 |
| **WNT7B** | 7.64771240026085e-06 | -2.27822080964847 |
| **FXYD4** | 0.000311176287626687 | -2.27524821580985 |
| **GREB1L** | 0.000222523298851297 | -2.27509493007749 |
| **SYT7** | 9.2711948620101e-05 | -2.26625522212487 |
| **TCIM** | 4.43902201777331e-06 | -2.2584649676776 |
| **CLMP** | 3.12295191830167e-07 | -2.25766486979281 |
| **TUFT1** | 1.39405401617417e-07 | -2.25431718873268 |
| **CHGB** | 0.00264264647992517 | -2.24961389007147 |
| **RSPO3** | 0.00130573141581004 | -2.23846718419452 |
| **KLRG2** | 0.000613128116078551 | -2.23572220555605 |
| **GADD45G** | 0.00169509523355929 | -2.23401150460328 |
| **DES** | 0.00302347866613234 | -2.23016977192817 |
| **PTGS2** | 0.00205023660387373 | -2.21958562076067 |
| **BTG2** | 7.80959259043415e-07 | -2.2087191389906 |
| **MTRNR2L3** | 0.00444095511455649 | -2.2031629498564 |
| **ABCA13** | 0.00350151257345116 | -2.19736686994579 |
| **LINGO1** | 9.14875585267353e-07 | -2.19239634005083 |
| **CLDN14** | 1.09822601560186e-06 | -2.19114148735201 |
| **MFSD4A** | 7.58121799544782e-07 | -2.19092517566415 |
| **PRKAR2B** | 1.09002429880365e-05 | -2.18131975604242 |
| **FAM234B** | 5.06115815827136e-07 | -2.18090064504048 |
| **MTRNR2L6** | 0.00609239508209476 | -2.17615695515383 |
| **ALDH1A3** | 0.000344454031863412 | -2.17302634401547 |
| **HSPB7** | 0.00111738152501391 | -2.1720607457863 |
| **FGF14** | 0.00049062404641869 | -2.17157878421872 |
| **GAD1** | 0.000116620524686268 | -2.16934408939867 |
| **ECRG4** | 0.0034814182289112 | -2.1449483359111 |
| **PHLDA1** | 6.68037221809415e-06 | -2.13941527207669 |
| **CHAC1** | 0.010103161875484 | -2.13851986408143 |
| **SLC12A1** | 3.76576498625312e-06 | -2.13801619233824 |
| **EDNRB** | 0.000346151904574876 | -2.12711738217088 |
| **CHST7** | 7.90049673373701e-05 | -2.1172470336806 |
| **AQP2** | 3.26060873258557e-06 | -2.10854723513272 |
| **RANBP3L** | 1.72681866257595e-06 | -2.10546903795652 |
| **ADAMTS8** | 0.00256421040695642 | -2.10522833434007 |
| **PTCH2** | 0.00417374128276761 | -2.09215412701155 |
| **LONRF1** | 1.43260114640696e-05 | -2.09154494187683 |
| **ANKRD2** | 0.0117394094764859 | -2.07662128160291 |
| **EGR3** | 0.000327130178558001 | -2.07414918267385 |
| **CA8** | 1.07556184958423e-06 | -2.07352903542197 |
| **SLC25A33** | 6.25735072561566e-07 | -2.06937633035253 |
| **SULT2B1** | 0.00363196472121632 | -2.06684759786445 |
| **SMIM38** | 0.0229787797754569 | -2.05994254045628 |
| **RYR2** | 0.0184358122411311 | -2.05993040602544 |
| **HS6ST2** | 0.000452022736761983 | -2.05452234691504 |
| **GATA6** | 2.0684509016798e-06 | -2.04406497889844 |
| **PPP1R1A** | 8.46572955398513e-05 | -2.03935443863689 |
| **ESRRB** | 0.00132052830108946 | -2.02077764330832 |
| **SLC19A2** | 0.000468429298521355 | -2.0191088229477 |
| **PRSS35** | 0.027015193848554 | -2.00921853002938 |
| **TMEM238L** | 0.01324398752111 | -2 |
| **VAT1L** | 4.73858062400888e-05 | -1.99514827057219 |
| **SLC38A11** | 0.00168667211982457 | -1.99131174693546 |
| **KLF9** | 2.2826416939215e-06 | -1.98648366184887 |
| **SLC4A7** | 3.24559611282711e-06 | -1.98635197839914 |
| **TIMP3** | 1.8697881158939e-05 | -1.97820729933365 |
| **FKBP5** | 2.25318423080403e-07 | -1.97611529588317 |
| **AQP4** | 0.0210716979314989 | -1.97605430437497 |
| **FMC1** | 0.00592180539386861 | -1.97450857233623 |
| **PLEKHS1** | 0.0222512370427513 | -1.96702267755373 |
| **EHF** | 0.000119688010572544 | -1.94552607901416 |
| **SPINK13** | 0.0423794420900396 | -1.94049898825134 |
| **ELF5** | 0.000355538705688296 | -1.93256232266275 |
| **FHL5** | 0.000801785784392185 | -1.92930627395558 |
| **SYBU** | 2.0082603350144e-06 | -1.92490956868037 |
| **PID1** | 0.00898921036097408 | -1.92110558884814 |
| **SLC5A3** | 9.75841478609931e-06 | -1.9210425215535 |
| **FOSL2** | 6.3538226687911e-05 | -1.8860576732261 |
| **NTF3** | 0.000675392446362903 | -1.88145269603482 |
| **NFATC2** | 0.000542701384350141 | -1.87998531155747 |
| **MRPS6** | 2.20345768222109e-05 | -1.86508000774309 |
| **RHOBTB3** | 2.65930936501222e-06 | -1.8637477564787 |
| **CCDC71L** | 0.00431967462648948 | -1.86238031255252 |
| **MT1M** | 0.0110753960801462 | -1.85432254480436 |
| **PRSS16** | 0.0197317228916116 | -1.85113661408552 |
| **SHISA3** | 8.16345276426918e-06 | -1.85022157166946 |
| **AQP3** | 0.0028339745118929 | -1.84532841514266 |
| **C21orf62** | 0.00219703450702514 | -1.84426888014044 |
| **GREM1** | 0.00354867339458652 | -1.8434096370593 |
| **AKAP6** | 9.61312614182985e-06 | -1.83769175741974 |
| **VCL** | 4.56913027080905e-06 | -1.83606838710262 |
| **ACTG2** | 0.0225403856657482 | -1.82505692379106 |
| **NUDT4** | 6.02209109103912e-06 | -1.82341218239277 |
| **AKR1B1** | 0.000117868680942107 | -1.82097396329047 |
| **LTBP1** | 0.000392235586126969 | -1.8175862953828 |
| **KIAA0825** | 0.00031501879544662 | -1.81672584387054 |
| **IRGM** | 0.0369195968559563 | -1.81537848735099 |
| **CALCA** | 0.0177649521842095 | -1.81262986409828 |
| **PDE3A** | 0.0237220224578366 | -1.80982112620046 |
| **RND3** | 4.79167500295102e-06 | -1.8026312581312 |
| **ADAMTS15** | 0.000900968393914357 | -1.80164456036909 |
| **TACC1** | 5.40719936130345e-06 | -1.78434631186761 |
| **NR4A2** | 0.00333333159471613 | -1.77379053450518 |
| **ASPN** | 0.0110387153261789 | -1.7734337612355 |
| **SIK1B** | 3.30427350302519e-06 | -1.75960844625789 |
| **RGS2** | 0.00343785602932596 | -1.75293560057611 |
| **SIK1** | 0.000260513791683906 | -1.74747088112654 |
| **IGFBP2** | 5.55874847896248e-06 | -1.73097397161996 |
| **SYN3** | 0.00102998617498219 | -1.7285936806918 |
| **MAFF** | 3.53165426322473e-06 | -1.72694008395271 |
| **MED12L** | 1.43882562685469e-05 | -1.72190738166028 |
| **SIAH3** | 0.0124710998157782 | -1.72122285762046 |
| **GALR1** | 0.0249059236764754 | -1.71959846219612 |
| **MLF1** | 1.00695919524816e-05 | -1.71927426834722 |
| **ANKRD37** | 0.0255920424788659 | -1.71620703399941 |
| **CAVIN2** | 3.729218364904e-06 | -1.71514431075777 |
| **PLAU** | 0.00490909635381853 | -1.71137009654727 |
| **ATP1B3** | 0.00424503728727362 | -1.70904024801591 |
| **TDGF1** | 0.0351636858144994 | -1.70290375809257 |
| **TMEM61** | 0.00702786429583404 | -1.70274412242762 |
| **SYNGR3** | 0.0470977337595049 | -1.69743722997957 |
| **GREM2** | 0.00260553851217336 | -1.69302224657861 |
| **CNN1** | 0.0122534646876418 | -1.68309872218747 |
| **GDF15** | 0.00122268187598218 | -1.67473729399087 |
| **CLDN19** | 0.0121270564962491 | -1.67442412809749 |
| **CEBPD** | 0.00231181966402507 | -1.67418048976007 |
| **HPSE2** | 2.41952811669183e-05 | -1.67225178467313 |
| **PTGER2** | 0.0283658349880827 | -1.66460288806303 |
| **NCAM1** | 0.0120418442048515 | -1.66245938938957 |
| **CYFIP2** | 1.11916724804561e-05 | -1.66078522706396 |
| **CALCR** | 0.00554697903118364 | -1.65556171649037 |
| **FAM43A** | 0.0240331928273834 | -1.64769825606912 |
| **ZNF331** | 0.00464698780757046 | -1.64713147867247 |
| **F2RL3** | 0.0326353526038242 | -1.63742992061529 |
| **SLC25A18** | 0.0251842902194092 | -1.63512529545925 |
| **PCDHB1** | 0.0429299556904268 | -1.63346101841235 |
| **ATP1A1** | 1.28745273598862e-05 | -1.63253770201418 |
| **AGTR1** | 0.00166259696859112 | -1.63231233914387 |
| **PFKFB3** | 3.66112766803742e-05 | -1.62767535308956 |
| **ELL2** | 0.00483438863230188 | -1.62110911225113 |
| **APOD** | 0.0410419507033955 | -1.61899267235862 |
| **COL26A1** | 0.00105697233807237 | -1.61811736075371 |
| **DNAJC19** | 0.0106112885451707 | -1.60700700004154 |
| **ITPRIP** | 0.00660415070946502 | -1.60523157615841 |
| **DPT** | 0.023662806170389 | -1.60508920057307 |
| **LAG3** | 0.0390286331937477 | -1.60072981657947 |
| **NELL1** | 0.00146243998083521 | -1.59443234355697 |
| **AKR1C1** | 2.20155522821267e-05 | -1.58151207947057 |
| **DNAH2** | 0.0129164384472539 | -1.57366246828874 |
| **USP2** | 0.00263687412874447 | -1.57167584071591 |
| **S1PR3** | 0.0121682826320399 | -1.56828375957453 |
| **INSYN2A** | 0.0272848094126269 | -1.56828375957453 |
| **SLC7A1** | 0.00698505106051368 | -1.56284768579458 |
| **CAPN6** | 0.0133509521185386 | -1.56075432987646 |
| **STC1** | 0.0305507906672594 | -1.55985971997146 |
| **USP53** | 2.75792024781455e-05 | -1.55058094290342 |
| **CCNB1IP1** | 0.000802527765146943 | -1.54422022111161 |
| **SHF** | 0.0374169468470157 | -1.53884015402239 |
| **RDH10** | 1.2629876987636e-05 | -1.53854373532584 |
| **SLC16A12** | 0.0039184943556176 | -1.52768438542945 |
| **SLCO1A2** | 0.0344120106112754 | -1.52758027858519 |
| **ZBTB16** | 0.00747712524441105 | -1.52243210144296 |
| **FOXP2** | 2.44307173698753e-05 | -1.51411635434986 |
| **GSTM3** | 0.00118209417850231 | -1.50831113556086 |
| **CSRNP1** | 1.88533674976414e-05 | -1.505559563542 |
| **GEM** | 0.0143212911567224 | -1.50480124529294 |
| **SEMA6D** | 0.00239596831706034 | -1.50214077329246 |

**Supplementary Table S2**. Upregulated and downregulated genes for the comparison between APS and SLE patients

| **UpRegulated genes, APS vs SLE** |  |  |
| --- | --- | --- |
| **gene_name** | **meta_p-value_APS_vs_SLE** | **log2_normalized_fold_change_APS_vs_SLE** |
| AREG | 0.00332841257534827 | 3.84728110647036 |
| GFAP | 0.00165522285264401 | 3.7742528270304 |
| ADGRG7 | 0.00167500118433846 | 3.30960134521193 |
| PGA3 | 0.0261862215085865 | 2.89481776330794 |
| PGA4 | 0.0263569472079687 | 2.87572966412206 |
| PGA5 | 0.0163091893230726 | 2.86249647625007 |
| CALY | 0.00178150976035158 | 2.59373271034889 |
| EREG | 0.0102969203046067 | 2.53637176310832 |
| MUC5AC | 0.00963402852959514 | 2.50153564972701 |
| MUC17 | 0.00721631048989127 | 2.49737010129724 |
| KRT17 | 0.00489360876856877 | 2.43382097495826 |
| BCAN | 0.00444741688874797 | 2.35535109642481 |
| APOB | 0.0173243633475884 | 2.33382177346862 |
| ASTL | 0.00930814196512485 | 2.31310424450036 |
| FCRL1 | 0.01103629400056 | 2.2786243106665 |
| GPR183 | 0.0124560071872624 | 2.07340093263807 |
| RAB44 | 0.000857814198235901 | 2.05889368905357 |
| MUC12 | 0.00132452836538376 | 2.00035495019022 |
| AC068775.1 | 0.00461655362564781 | 1.99652780809319 |
| BLK | 0.00858023527026388 | 1.9960164860815 |
| EPHA8 | 0.0043008994403365 | 1.99435343685886 |
| NGB | 0.00447570034672903 | 1.97727992349992 |
| CCR7 | 0.0088230796866405 | 1.97174173995849 |
| AC008397.1 | 0.0265621689539768 | 1.96238570820022 |
| GOLGA6L2 | 0.00617245861463244 | 1.9584208962486 |
| SYNDIG1L | 0.0258741935232384 | 1.95735566259151 |
| AC067752.1 | 0.00313081366794263 | 1.95293796312796 |
| CLEC17A | 0.00255261202793848 | 1.95197848513706 |
| KLRC2 | 0.00762182226869272 | 1.95128471496697 |
| TERT | 0.00113514777230594 | 1.94811325833503 |
| CEACAM20 | 0.0150344062568017 | 1.93411206434354 |
| CD1C | 0.0311192769359639 | 1.90689059560852 |
| SCEL | 0.00524395475646736 | 1.90321493746512 |
| MS4A1 | 0.0142047804568323 | 1.89058878327942 |
| TMC3 | 0.00336368009894441 | 1.877744249949 |
| OTOF | 0.00568399897332968 | 1.87586369998789 |
| SCML4 | 0.00630114699035856 | 1.83181660379528 |
| CXCR4 | 0.0117490662852412 | 1.80374037844047 |
| RBFOX3 | 0.00170433887448377 | 1.80343010882523 |
| NR4A3 | 0.0144827518990723 | 1.78560252132985 |
| SERPINA3 | 0.0404976027613541 | 1.77683005504415 |
| IL1R2 | 0.00776795340498419 | 1.76081233612057 |
| TNFRSF13C | 0.0197929234607077 | 1.74461916670964 |
| CD69 | 0.0273286051623262 | 1.74024072619907 |
| LHX2 | 0.00884783052990528 | 1.73696559416621 |
| CCDC155 | 0.0088230968633275 | 1.73256041504621 |
| GAGE12F | 0.0169556765918214 | 1.73001283352444 |
| MEP1A | 0.00721825632007298 | 1.72354507840388 |
| IL7R | 0.00800188772424074 | 1.71197121469934 |
| PAX5 | 0.00218625952297372 | 1.71171327701986 |
| CEACAM5 | 0.0486083235648861 | 1.70659194512269 |
| FCAR | 0.0029029887637023 | 1.70274987882829 |
| PRR20C | 0.0212746628408173 | 1.70207080372002 |
| MUC13 | 0.0162636577998785 | 1.68974643849356 |
| LYPD8 | 0.014482607121961 | 1.67151253267759 |
| NSG1 | 0.00644089986615097 | 1.63400840381838 |
| GABRA5 | 0.0141943790562109 | 1.62910960440554 |
| RHEX | 0.0148302872815055 | 1.60371375342672 |
| DUX4 | 0.0221570850693084 | 1.59578436920117 |
| FLT3 | 0.0123243263986378 | 1.58582924453869 |
| CEACAM6 | 0.0161857654491203 | 1.56768450928932 |
| CXCL9 | 0.0334057225527965 | 1.55541822510714 |
| GLDN | 0.00105130041840606 | 1.54328446947129 |
| IL18RAP | 0.010166121849667 | 1.54257285393754 |
| CACNA1I | 0.00610828131696563 | 1.54247916939964 |
| MYB | 0.00777192818850209 | 1.54035161402086 |
| DYTN | 0.0218169518849682 | 1.5398331252315 |
| KCNK17 | 0.0038135885693707 | 1.53951952995999 |
| OLFM4 | 0.0430112201485424 | 1.52436590984817 |
| CASP14 | 0.01669365592699 | 1.52356195605701 |
| CEMIP | 0.00802215499057038 | 1.51470537856097 |
| TREM1 | 0.0455484427765542 | 1.51362371900089 |
| RIMS1 | 0.00436591474572714 | 1.51193506942618 |
| CENPM | 0.00227079097683825 | 1.50955509806313 |
| TMPRSS6 | 0.0120179468279189 | 1.50250034052918 |

| **DownRegulated APS vs SLE** |  |  |
| --- | --- | --- |
| **gene_name** | **meta_p-value_APS_vs_SLE** | **log2_normalized_fold_change_APS_vs_SLE** |
| **MYH2** | 2.69375830226976e-12 | -9.09714859914784 |
| **MYH1** | 4.32223521414584e-11 | -8.22814358751637 |
| **TTN** | 2.52231579158786e-10 | -7.30669692192016 |
| **CKM** | 3.73366355527973e-09 | -6.74264196026996 |
| **TNNT1** | 7.50241857606805e-08 | -6.07479238420072 |
| **MYH6** | 2.3450762192848e-08 | -6.03155806850265 |
| **DES** | 1.86490555425619e-08 | -5.76128527336162 |
| **NEB** | 1.33190052852307e-09 | -5.75477340551737 |
| **MYBPC1** | 1.67615196066001e-08 | -5.65468791003623 |
| **NRAP** | 1.51991426083524e-07 | -5.45856422342651 |
| **TNNT3** | 9.86408549175643e-11 | -5.3675182118612 |
| **IGFN1** | 2.0630997253131e-05 | -5.13911187959197 |
| **TNNC2** | 0.000105477913064699 | -4.99656091603323 |
| **MB** | 1.3166566475677e-09 | -4.97205140869811 |
| **EEF1A2** | 0.000379417269115607 | -4.6828098241193 |
| **XIRP2** | 4.47178033184189e-08 | -4.49859965631198 |
| **TNNI1** | 0.000218627634099677 | -4.38461870888327 |
| **MYBPC2** | 0.000562114498512379 | -4.32918825779084 |
| **ATP2A1** | 0.000650105564967804 | -4.24492145035683 |
| **ACTN3** | 0.00236828691937137 | -4.05856389249461 |
| **KLHL41** | 0.00158918317638499 | -3.88539687836014 |
| **CASQ1** | 0.00259257711633095 | -3.87902380217557 |
| **ACTN2** | 3.47203808445132e-05 | -3.81134520129938 |
| **MYOZ1** | 0.0013977005276211 | -3.75386239271708 |
| **FLNC** | 2.57289098465387e-06 | -3.65642009600005 |
| **MYOT** | 0.00284977066104486 | -3.54823446747006 |
| **PYGM** | 0.00145122172124172 | -3.51196667701528 |
| **MYLK2** | 0.017924461228348 | -3.46276622975907 |
| **DDIT4L** | 0.00132424682461046 | -3.35495689527483 |
| **ADIPOQ** | 0.0242806111564884 | -3.3109688828975 |
| **LMOD3** | 0.0188828719535032 | -3.11484292633876 |
| **SMTNL1** | 0.00931353532000197 | -3.08859392326899 |
| **MYOC** | 0.0259730886469661 | -3.00523664836615 |
| **MYH8** | 0.00211847820078529 | -2.99604198643132 |
| **ANKRD2** | 0.0040984679143192 | -2.91537603585301 |
| **CACNA1S** | 0.00939784076403371 | -2.91130451698375 |
| **RPL3L** | 0.0290298233559359 | -2.90731799749015 |
| **HSPB7** | 0.00459096435823096 | -2.87026607039598 |
| **HHATL** | 0.0173532550288203 | -2.85663582482882 |
| **CCL8** | 0.0186904502170152 | -2.83650126771712 |
| **RYR1** | 0.00183151439434104 | -2.81675855390243 |
| **GC** | 9.52923204732805e-05 | -2.80265132963983 |
| **SMPX** | 0.0356894135579109 | -2.80145432061792 |
| **ENO3** | 0.00223696546399781 | -2.79564942140532 |
| **CSRP3** | 0.0224290180430068 | -2.79422565011397 |
| **RBM24** | 0.0120045853690886 | -2.78197277122495 |
| **HSD17B2** | 0.0269945131257727 | -2.77003074317718 |
| **ACTC1** | 0.0486787812801612 | -2.70555264153474 |
| **AMPD1** | 0.0127008087916646 | -2.70043971814109 |
| **PERM1** | 0.0116448826659699 | -2.67807190511264 |
| **FBXO40** | 0.0238546074535025 | -2.66812910186506 |
| **PRG4** | 0.0436831733852742 | -2.65207669657969 |
| **SLC7A13** | 0.000608747604454411 | -2.64769825606912 |
| **TRIM54** | 0.010289236316718 | -2.6445349453934 |
| **TRDN** | 0.00712355879070263 | -2.63920208861922 |
| **SYNPO2L** | 0.025366268497035 | -2.5376567859428 |
| **MYPN** | 0.0253601354763807 | -2.51519222241698 |
| **SH3GL3** | 0.027674846559435 | -2.50303064564116 |
| **SMYD1** | 0.0376012320202924 | -2.43888424123321 |
| **ECRG4** | 0.00129911435707107 | -2.39376888252519 |
| **INS-IGF2** | 0.0076724918432586 | -2.34714974037717 |
| **IGF2** | 0.00345849612301867 | -2.33168373620604 |
| **AC132217.2** | 0.00358828991320197 | -2.31429782395483 |
| **APOD** | 0.00722027397683407 | -2.29996097483215 |
| **TPM2** | 0.000426516318301655 | -2.29684829834398 |
| **MYH13** | 0.0403947506183164 | -2.20707799985978 |
| **ASB5** | 0.0267214658422071 | -2.17989317053355 |
| **KLHL31** | 0.0341299812589587 | -2.14284684482424 |
| **CILP** | 0.0109798580931365 | -2.11977735485356 |
| **G0S2** | 2.58290068168175e-06 | -2.10116946657838 |
| **PROX1** | 0.00263869422096458 | -2.09752266889414 |
| **C10orf71** | 0.0434410890105479 | -2.09276520898465 |
| **EGF** | 0.000452037570766854 | -2.08975360323896 |
| **SLC4A11** | 0.00890494407092002 | -2.08814656825953 |
| **DACH2** | 0.0191964894601961 | -2.06972747865779 |
| **PDLIM3** | 0.0280331297125826 | -2.00375865182003 |
| **MYH7** | 2.10537600755208e-13 | -10.1237605027637 |
| **ACTA1** | 2.34976913684883e-13 | -10.0051031922023 |
| **MYOZ2** | 0.00871150885815084 | -1.98672357157433 |
| **HSPA2** | 0.00102774933386684 | -1.97538406146424 |
| **MEOX2** | 0.0184617095527163 | -1.93421992536119 |
| **CALML3** | 0.0158741350218721 | -1.90195702709015 |
| **STAC3** | 0.0267366246467136 | -1.8903258796595 |
| **SMIM38** | 0.0243421861498771 | -1.83922669692722 |
| **PLIN4** | 0.0233225154421505 | -1.8307696493973 |
| **EMILIN3** | 0.0442305955976969 | -1.82312223791592 |
| **MYOZ3** | 0.0421426388253194 | -1.81855312866247 |
| **UMOD** | 0.000112457016624564 | -1.81242895622693 |
| **MYH3** | 0.018363532938532 | -1.80418067426246 |
| **OLFM1** | 0.0443897483507322 | -1.80110817072841 |
| **FABP5** | 0.0222443923374691 | -1.77928415647094 |
| **TXLNB** | 0.0415758067883146 | -1.75575126893451 |
| **THBS4** | 0.0149224987634327 | -1.75157338965274 |
| **SLC2A4** | 0.0204721264743049 | -1.73568262828273 |
| **CAVIN4** | 0.0423693797494164 | -1.66965966529644 |
| **DPT** | 0.00515811307893323 | -1.6550885871118 |
| **SPTB** | 0.0177152741997611 | -1.63951711025002 |
| **SYT1** | 0.0489849701900388 | -1.63866015081742 |
| **RBP4** | 0.033560122237277 | -1.6369859461416 |
| **PPP1R1A** | 0.00321160376683563 | -1.63098066963905 |
| **OR2T11** | 0.01649785523615 | -1.62173199586915 |
| **CMYA5** | 0.0185718547804767 | -1.59584074244592 |
| **TMEM207** | 0.0248350187640215 | -1.58431685513379 |
| **MUC15** | 0.0150022688398914 | -1.57919665502983 |
| **HRC** | 0.00979854221945633 | -1.57878392579203 |
| **PGAM2** | 0.0476566735298535 | -1.57337452644594 |
| **MYH7B** | 0.0211235831509336 | -1.56793054741928 |
| **STC1** | 0.0333190146861042 | -1.55985971997146 |
| **PLAU** | 0.00921249085178973 | -1.54992479577226 |
| **ATP1A2** | 0.0381509667551265 | -1.51614730832787 |
| **ARPP21** | 0.0142128778374968 | -1.51470894950304 |

**Supplementary Table S3.** Enrichment analysis for the comparison between patients with APS and healthy controls

|  | | | | |
| --- | --- | --- | --- | --- |
| **p_value** | **intersection_size** | **precision** | **term_id** | **term_name** |
| 0.00832405804355686 | 2 | 0.125 | CORUM:6107 | proMBP-angiotensinogen-c3dg complex |
| 0.0000000000101422525432024 | 78 | 0.322 | GO:0002376 | immune system process |
| 0.000000000548797138964162 | 61 | 0.252 | GO:0006955 | immune response |
| 0.00000000120371778182702 | 57 | 0.236 | GO:0006952 | defense response |
| 0.00000000136943281977691 | 73 | 0.302 | GO:0009605 | response to external stimulus |
| 0.00000000230158601393437 | 51 | 0.211 | GO:0002682 | regulation of immune system process |
| 0.00000000616336402479399 | 39 | 0.161 | GO:0045321 | leukocyte activation |
| 0.00000000722076903071048 | 42 | 0.174 | GO:0001775 | cell activation |
| 0.00000000777249969689216 | 76 | 0.314 | GO:0051239 | regulation of multicellular organismal process |
| 0.00000000951150449697934 | 54 | 0.223 | GO:0044419 | biological process involved in interspecies interaction between organisms |
| 0.0000000175478052110241 | 51 | 0.211 | GO:0009607 | response to biotic stimulus |
| 0.0000000247410552871877 | 50 | 0.207 | GO:0051707 | response to other organism |
| 0.0000000265587018179356 | 50 | 0.207 | GO:0043207 | response to external biotic stimulus |
| 0.0000000893226387384324 | 30 | 0.124 | GO:0002252 | immune effector process |
| 0.00000010862896116622 | 39 | 0.161 | GO:0002684 | positive regulation of immune system process |
| 0.000000188789381758859 | 33 | 0.136 | GO:0046649 | lymphocyte activation |
| 0.000000319299406556261 | 152 | 0.628 | GO:0050896 | response to stimulus |
| 0.00000054439376929927 | 36 | 0.149 | GO:0045087 | innate immune response |
| 0.00000102531714822338 | 34 | 0.140 | GO:0050776 | regulation of immune response |
| 0.0000016562594895746 | 120 | 0.496 | GO:0007154 | cell communication |
| 0.00000214679123203682 | 40 | 0.165 | GO:0098542 | defense response to other organism |
| 0.0000023204007837733 | 134 | 0.554 | GO:0032501 | multicellular organismal process |
| 0.0000026665627872834 | 58 | 0.240 | GO:0048584 | positive regulation of response to stimulus |
| 0.00000298230364975442 | 118 | 0.488 | GO:0023052 | signaling |
| 0.00000365538569658841 | 50 | 0.207 | GO:0051049 | regulation of transport |
| 0.0000065582099204151 | 14 | 0.058 | GO:0045807 | positive regulation of endocytosis |
| 0.000013680620990289 | 31 | 0.128 | GO:0006954 | inflammatory response |
| 0.0000163802742732087 | 10 | 0.041 | GO:0050766 | positive regulation of phagocytosis |
| 0.0000184795053687004 | 24 | 0.099 | GO:0060627 | regulation of vesicle-mediated transport |
| 0.0000212050030984946 | 17 | 0.070 | GO:0002699 | positive regulation of immune effector process |
| 0.0000312691631575473 | 16 | 0.066 | GO:0006909 | phagocytosis |
| 0.0000374952979195777 | 11 | 0.045 | GO:0050764 | regulation of phagocytosis |
| 0.0000380110984558122 | 25 | 0.103 | GO:0002694 | regulation of leukocyte activation |
| 0.0000476443251345091 | 26 | 0.107 | GO:0050865 | regulation of cell activation |
| 0.0000479125318506326 | 64 | 0.264 | GO:0010033 | response to organic substance |
| 0.0000614762089559217 | 108 | 0.446 | GO:0007165 | signal transduction |
| 0.0000892072631485801 | 62 | 0.256 | GO:0070887 | cellular response to chemical stimulus |
| 0.000128775106074613 | 63 | 0.260 | GO:0007166 | cell surface receptor signaling pathway |
| 0.000173867097818042 | 44 | 0.182 | GO:0051240 | positive regulation of multicellular organismal process |
| 0.000192244756446077 | 24 | 0.099 | GO:0032103 | positive regulation of response to external stimulus |
| 0.000196691350757091 | 19 | 0.079 | GO:0002697 | regulation of immune effector process |
| 0.000199257975496025 | 80 | 0.331 | GO:0042221 | response to chemical |
| 0.000221205525945124 | 52 | 0.215 | GO:0032879 | regulation of localization |
| 0.000250767696028904 | 19 | 0.079 | GO:0002443 | leukocyte mediated immunity |
| 0.000264593401932617 | 31 | 0.128 | GO:0098657 | import into cell |
| 0.000267694214374521 | 20 | 0.083 | GO:0009615 | response to virus |
| 0.000307283046872525 | 26 | 0.107 | GO:0050778 | positive regulation of immune response |
| 0.000405063977168681 | 41 | 0.169 | GO:0007155 | cell adhesion |
| 0.000425301434640047 | 16 | 0.066 | GO:0030100 | regulation of endocytosis |
| 0.000452958233275639 | 78 | 0.322 | GO:0048583 | regulation of response to stimulus |
| 0.000519216210238034 | 89 | 0.368 | GO:0006810 | transport |
| 0.00056640442257562 | 49 | 0.202 | GO:0071310 | cellular response to organic substance |
| 0.000614015891175111 | 102 | 0.421 | GO:0048522 | positive regulation of cellular process |
| 0.000620431492236072 | 26 | 0.107 | GO:0006897 | endocytosis |
| 0.000774735805724484 | 123 | 0.508 | GO:0051716 | cellular response to stimulus |
| 0.000843659387823854 | 22 | 0.091 | GO:0042110 | T cell activation |
| 0.000858806060288564 | 30 | 0.124 | GO:0098609 | cell-cell adhesion |
| 0.000961865481969664 | 77 | 0.318 | GO:0006950 | response to stress |
| 0.00116932479267687 | 41 | 0.169 | GO:0016192 | vesicle-mediated transport |
| 0.00120319852341477 | 108 | 0.446 | GO:0048518 | positive regulation of biological process |
| 0.00124380051273052 | 32 | 0.132 | GO:0032101 | regulation of response to external stimulus |
| 0.00148981121807515 | 18 | 0.074 | GO:0050900 | leukocyte migration |
| 0.00152876814961034 | 29 | 0.120 | GO:0051050 | positive regulation of transport |
| 0.00188465424098888 | 26 | 0.107 | GO:0001817 | regulation of cytokine production |
| 0.00217748182652819 | 26 | 0.107 | GO:0001816 | cytokine production |
| 0.00245380803722632 | 9 | 0.037 | GO:0048525 | negative regulation of viral process |
| 0.00261907864635191 | 20 | 0.083 | GO:0051249 | regulation of lymphocyte activation |
| 0.00263117615742907 | 15 | 0.062 | GO:0002449 | lymphocyte mediated immunity |
| 0.00309358277887333 | 89 | 0.368 | GO:0051234 | establishment of localization |
| 0.00373733875261157 | 40 | 0.165 | GO:0055085 | transmembrane transport |
| 0.00516763411871099 | 17 | 0.070 | GO:0050867 | positive regulation of cell activation |
| 0.00556092356448065 | 15 | 0.062 | GO:0046651 | lymphocyte proliferation |
| 0.00601107084165034 | 7 | 0.029 | GO:0045071 | negative regulation of viral genome replication |
| 0.00656706291188105 | 4 | 0.017 | GO:0015747 | urate transport |
| 0.00732814620335889 | 15 | 0.062 | GO:0032943 | mononuclear cell proliferation |
| 0.00888331689216222 | 17 | 0.070 | GO:0007159 | leukocyte cell-cell adhesion |
| 0.00922574151869323 | 15 | 0.062 | GO:0019058 | viral life cycle |
| 0.010882776340804 | 28 | 0.116 | GO:0046903 | secretion |
| 0.010992385306238 | 18 | 0.074 | GO:0031349 | positive regulation of defense response |
| 0.0133170011495119 | 16 | 0.066 | GO:0002696 | positive regulation of leukocyte activation |
| 0.0137745029180527 | 16 | 0.066 | GO:0050863 | regulation of T cell activation |
| 0.0172016708458434 | 15 | 0.062 | GO:0051607 | defense response to virus |
| 0.0178207865793441 | 15 | 0.062 | GO:0140546 | defense response to symbiont |
| 0.0198192968242556 | 23 | 0.095 | GO:0002250 | adaptive immune response |
| 0.0202977956265782 | 37 | 0.153 | GO:0009967 | positive regulation of signal transduction |
| 0.0225392382769947 | 14 | 0.058 | GO:0002460 | adaptive immune response based on somatic recombination of immune receptors built from immunoglobulin superfamily domains |
| 0.023303914394418 | 9 | 0.037 | GO:0072676 | lymphocyte migration |
| 0.0245798174372259 | 26 | 0.107 | GO:0140352 | export from cell |
| 0.0259500975766656 | 33 | 0.136 | GO:0006811 | monoatomic ion transport |
| 0.0260602154398291 | 15 | 0.062 | GO:0070661 | leukocyte proliferation |
| 0.0284984800756775 | 18 | 0.074 | GO:0019221 | cytokine-mediated signaling pathway |
| 0.0288268738044657 | 15 | 0.062 | GO:0002833 | positive regulation of response to biotic stimulus |
| 0.0309436987208671 | 40 | 0.165 | GO:0010647 | positive regulation of cell communication |
| 0.0317427229083677 | 8 | 0.033 | GO:0034109 | homotypic cell-cell adhesion |
| 0.0317925338025428 | 18 | 0.074 | GO:0002831 | regulation of response to biotic stimulus |
| 0.0334597348482054 | 26 | 0.107 | GO:0030001 | metal ion transport |
| 0.0359806004060267 | 16 | 0.066 | GO:0045088 | regulation of innate immune response |
| 0.0402715363773534 | 12 | 0.050 | GO:0050670 | regulation of lymphocyte proliferation |
| 0.0421352577154489 | 5 | 0.021 | GO:0034369 | plasma lipoprotein particle remodeling |
| 0.0421352577154489 | 5 | 0.021 | GO:0034368 | protein-lipid complex remodeling |
| 0.0427215767443396 | 26 | 0.107 | GO:0034097 | response to cytokine |
| 0.0448450468489334 | 23 | 0.095 | GO:0031347 | regulation of defense response |
| 0.0475067257375324 | 12 | 0.050 | GO:0032944 | regulation of mononuclear cell proliferation |
| 0.0478106900181109 | 11 | 0.045 | GO:0071674 | mononuclear cell migration |
| 0.0480027802624628 | 93 | 0.384 | GO:0051179 | localization |
| 0.0499232146791529 | 18 | 0.074 | GO:0002253 | activation of immune response |
| 0.00703809043381687 | 9 | 0.076 | KEGG:04650 | Natural killer cell mediated cytotoxicity |
| 0.0223878279488382 | 7 | 0.059 | KEGG:04610 | Complement and coagulation cascades |
| 0.0240565725233449 | 7 | 0.059 | KEGG:05150 | Staphylococcus aureus infection |
| 0.0499223618574666 | 7 | 0.059 | KEGG:04061 | Viral protein interaction with cytokine and cytokine receptor |
| 0.000682444254944048 | 9 | 0.053 | REAC:R-HSA-909733 | Interferon alpha/beta signaling |
| 0.00200125548889068 | 57 | 0.333 | REAC:R-HSA-168256 | Immune System |
| 0.00483776176173266 | 21 | 0.123 | REAC:R-HSA-500792 | GPCR ligand binding |
| 0.0262535956356282 | 16 | 0.094 | REAC:R-HSA-373076 | Class A/1 (Rhodopsin-like receptors) |
| 0.0321568822097549 | 3 | 0.018 | REAC:R-HSA-211916 | Vitamins |
